# Rationale and Design of the PAIRED Trial: Partnered Dance Aerobic Exercise as a Neuroprotective, Motor, and Cognitive Intervention in Parkinson's Disease

**DOI:** 10.3389/fneur.2020.00943

**Published:** 2020-10-09

**Authors:** Madeleine E. Hackney, Allison A. Bay, Jordan M. Jackson, Joe R. Nocera, Venkatagiri Krishnamurthy, Bruce Crosson, Marian L. Evatt, Jason Langley, Xiangqin Cui, J. Lucas McKay, Daniel E. Huddleston

**Affiliations:** ^1^Center for Visual and Neurocognitive Rehabilitation, Atlanta VA, Decatur, GA, United States; ^2^Division of Geriatrics and Gerontology, Department of Medicine, Emory University School of Medicine, Atlanta, GA, United States; ^3^Department of Rehabilitation Medicine, Emory University School of Medicine, Atlanta, GA, United States; ^4^Emory University School of Nursing, Atlanta, GA, United States; ^5^Birmingham/Atlanta VA Geriatric Research Education and Clinical Center, Atlanta, GA, United States; ^6^Emory University Rollins School of Public Health, Atlanta, GA, United States; ^7^Emory University School of Medicine Department of Neurology, Atlanta, GA, United States; ^8^Georgia State University Department of Psychology, Atlanta, GA, United States; ^9^Health and Rehabilitation Science, University of Queensland, Brisbane, QLD, Australia; ^10^Center for Advanced Neuroimaging, University of California, Riverside, Riverside, CA, United States; ^11^Department of Biomedical Engineering, Emory University School of Medicine, Atlanta, GA, United States

**Keywords:** Parkinson's disease, clinical trial, movement, dance, functional neuroimaging, substantia nigra, exercise, walking

## Abstract

Parkinson's disease (PD), an intractable condition impairing motor and cognitive function, is imperfectly treated by drugs and surgery. Two priority issues for many people with PD are OFF-time and cognitive impairment. Even under best medical management, three-fourths of people with PD experience “OFF-time” related to medication-related motor fluctuations, which severely impacts both quality of life and cognition. Cognitive deficits are found even in newly diagnosed people with PD and are often intractable. Our data suggest that partnered dance aerobic exercise (PDAE) reduces OFF-time on the Movement Disorders Society Unified Parkinson Disease Rating Scale-IV (MDS-UPDRS-IV) and ameliorates other disease features, which motivate the PAIRED trial. PDAE provides AE during an improvisational, cognitively engaging rehabilitative physical activity. Although exercise benefits motor and cognitive symptoms and may be neuroprotective for PD, studies using robust biomarkers of neuroprotection in humans are rare. We propose to perform a randomized, controlled trial in individuals with diagnosed mild–moderate PD to compare the efficacy of PDAE vs. walking aerobic exercise (WALK) for OFF-time, cognition, and neuroprotection. We will assess neuroprotection with neuromelanin-sensitive MRI (NM-MRI) and iron-sensitive (R2^*^) MRI sequences to quantify neuromelanin loss and iron accumulation in substantia nigra pars compacta (SNc). We will use these biomarkers, neuromelanin loss, and iron accumulation, as tools to chart the course of neurodegeneration in patients with PD who have undergone long-term (16 months) intervention. We will randomly assign 102 individuals with mild–moderate PD to 16 months of PDAE or WALK. The 16-month intervention period will consist of Training (3 months of biweekly sessions) and Maintenance (13 months of weekly sessions) phases. We will assess participants at baseline, 3 months (immediately post-Training), and 16 months (immediately post-Maintenance) for OFF-time and behaviorally and physiologically measured cognition. We will acquire NM-MRI and R2^*^ imaging data at baseline and 16 months to assess neuroprotection. We will (1) examine effects of Training and Maintenance phases of PDAE vs. WALK on OFF-time, (2) compare PDAE vs. WALK at 3 and 16 months on behavioral and functional MRI (fMRI) measures of spatial cognition, and (3) compare PDAE vs. WALK for effects on rates of neurodegeneration.

## Introduction

Parkinson's disease (PD) impairs motor and non-motor functions and affects up to 2% of those aged 65 or more years. Annual costs associated with PD, including Medicare and Social Security, are around $25 billion annually in the United States (1). PD is almost ubiquitously treated with levodopa, a drug with side effects including motor fluctuations and dyskinesia, resulting in reduced sleep quality, difficulty with activities of daily living (ADLs), anxiety about drug effect, being immobilized and experiencing pain, and compromising quality of life (QOL) (2). Cognition plays a crucial role in motor function and functional ADLs, but one or more cognitive domains are impaired in up to 80% of patients beyond 5 years with PD (3). Although research supports several exercise principles that address motor issues, relationships between exercise principles and cognitive benefits in PD are less clear. Unfortunately, PD drugs and surgery imperfectly address motor and non-motor function for most people with PD, with decreasing efficacy of medications over time as this neurodegenerative disease progresses (2, 4).

Approximately 75% of people with PD experience “OFF-time” when medications do not work properly, and medication-related motor fluctuations (MRMF) result. MRMF adversely impact QOL. OFF-time is measured by part IV of the Movement Disorders Society Unified Parkinson's Disease Rating Scale (MDS-UPDRS) (2). Annually, 10% of patients develop MRMF and response failures when no symptomatic benefit occurs after taking levodopa (5). Long-term treatment results in MRMF in most patients (6). Within 4–6 years of levodopa treatment, MRMF develop in about 40% of PD patients, and after >10 years, nearly 90% of patients develop dyskinesias (7). Reducing dopaminergic therapy can not only improve fluctuations but may also increase PD symptoms (8, 9) or delay the “on” state. A total response failure accounted for more than 60% of daily OFF-time periods among 327 patients with advanced PD (10). Evidence suggests that exercise therapies play an ancillary role in drug efficacy, yet relationships between exercise modalities and OFF-time in PD have not been explicitly studied. Abundant evidence supports exercise benefits for people with PD, yet almost no research has targeted those who experience OFF-time.

People with PD report wanting reduced MRMF/OFF-time and 24-h symptom relief (11). Painful OFF-time and cognitive impairment become more common as PD neurodegeneration progresses (12). Using Level II criteria from Movement Disorders Society (MDS) task force on cognitive impairment, half of PD patients have cognitive impairment within 3 years of diagnosis (13). In individuals with PD for 5 years, nearly 80% have comorbid cognitive impairment (3). Levodopa only partially treats cognitive impairment (14). PD-related deficits in spatial attention, spatial location, and perception of optic flow (15) cause orientation disability and impair mobility (16). Spatial cognition is supported by brain structures vulnerable to normal cerebral aging (16) and PD neurodegenerative processes (15). Impaired attention and executive function also result in impaired mobility, dual tasking problems, postural-gait disturbances, and falls (17, 18). MRMF are significant predictors of pain: OFF-time episodes promote reduced pain thresholds (19) and worsening pain (19).

Exercise is increasingly touted as being helpful for motor and cognitive symptom relief (20) and may be neuroprotective (21). Our hypothesis, based on preliminary data, is that exercise reduces PD patient OFF-time. Exercise can improve dopamine efficiency: animals that exercised possessed less of the dopamine transporter; dopamine stayed in their synapses longer, and cells receiving the dopamine signal had more binding sites on the D2 receptor for dopamine and could receive a stronger signal (22). In a cohort of naive patients within 1 year of diagnosis and not on any medications, positron emission tomography (PET) showed that exercise increased expression of D2 receptors (23). Thus, exercise may impact the patient experience of drug efficacy by reducing patient OFF-time.

Previous small studies suggested that exercise had a null or variable impact on levodopa plasma absorption rates. Mouradian et al. (24) found no effect of one bout of acute treadmill training on the plasma levels of levodopa or antiparkinsonian effects in a study of four men optimally treated with levodopa (24). In 10 patients, Carter et al. (25) demonstrated that stationary cycling resulted in a patient-dependent increase or decrease in levodopa absorption (25). However, exercise may increase clinical efficacy of levodopa via other mechanisms. Reuter et al. (26) noted a trend toward better levodopa absorption and decreased dyskinesias under exercise conditions compared to rest in 12 patients (26). In a study of 12 male patients, levodopa plasma behavior did not differ between rest and exercise days, but motor response was better 120 and 150 min after levodopa intake on exercise days (27). However, these early studies were conducted in only patients without OFF-time. Therefore, the findings do not generalize to the impact of chronic exercise on OFF-time for patients who regularly experience this debilitating problem. This project will evaluate whether exercise reduces OFF-time in PD patients who regularly suffer MRMF.

Short, high-volume to long-term programs have ameliorated MRMF (28). In a 12-week study, effects of general strength/fitness exercise and levodopa were complementary, improving muscle force, UPDRS scores, and mobility (29). Frazzitta et al. (9) noted that scores on UPDRS section IV, which assays OFF-time and MRMF, improved after 4 weeks of intensive physiotherapy. Moreover, 6-month and 1-year follow-up scores on the UPDRS-III (clinical motor examination) were better than the admission scores although the therapy had been discontinued several months prior (9). It is plausible, then, that if such an effect was examined after 4 weeks of therapy, the effects would have been even stronger had the training continued in a less intense maintenance program.

Exercise also benefits cognition in PD. Uc et al. (30) demonstrated improved executive function in 43 PD patients (30). Duchesne et al. (31) showed improved inhibition and motor skill learning following AE in early PD (31). David et al. (32) reported that resistance training improved attention and working memory in 38 mild-to-moderately affected PD participants (32). Studies now examine physical and cognitive training delivered simultaneously or in close series to determine best treatments for cognitive impairment (33). Consistent with animal literature, dual cognitive/physical challenge may be most effective in inducing beneficial, durable effects on the brain's structure and function (34). This project will also evaluate the effects of dual cognitive/physical challenge and its effects on neural activity.

Evidence of improved motor and cognitive behavioral function with correlating neural-level changes and reduced OFF-time suggests that exercise may be neuroprotective in humans with PD **[**21, 35]. AE improves function and structure of the central nervous system (CNS) in older adults (35). AE is also associated with improved hippocampal function in older adults (36) and spared age-related deterioration of white matter tracts (37). Exercise in humans and rats increases brain-derived neurotrophic factor, insulin-like growth factors, nerve growth factor, and vascular endothelial growth factor (35). Only animal studies have provided evidence for exercise-induced neuroplasticity of corticostriatal circuits affected in PD. Exercise has been implicated in modulating dopamine neurotransmission, altering synaptogenesis, and increasing cerebral blood flow (22). In a PD rat model, AE increased activation in ventral striatum and pallidum, while it improved motor deficits (38). With physiological biomarkers, we aim to show that exercise slows neurodegeneration in humans with PD. Novel imaging tools and sequences are available and allow us to elucidate this relationship if it exists.

We aim to show that exercise slows neurodegeneration in humans with PD. Clinicians currently primarily monitor disease progression using the MDS-UPDRS assessment, a non-reliable measure of neurodegeneration in substantia nigra (SN) and other areas damaged by PD. The physiological magnetic resonance imaging (MRI) measurement of neuromelanin (NM) loss and iron accumulation can more accurately reflect disease progression over time. At PD diagnosis, 50% of the melanized dopamine neurons (NM) in the SN pars compacta (SNc) have died (39). The SNc and SN pars reticulata (SNr) are predominantly NM and iron containing, respectively (40).

NM loss is accompanied by iron accumulation in astrocytes, microglia, and neurons. Neurodegenerative changes in NM and iron in the SNc can be quantified *in vivo* with NM-MRI (41). NM-MRI also detects progressive loss of NM as PD advances (42). Our published studies support the use of these measures. Using an automated, reproducible segmentation approach, our methods can detect significant PD-associated NM loss, iron accumulation, and microstructural damage in SNc (43–45). The NM-MRI approach proposed in this study achieves high scan–rescan reproducibility (ICC = 0.96) (45), generates more contrast and deposits less energy than other approaches (41), and has improved signal-to-noise ratio (SNR) in SNc compared to other approaches (41, 46). The NM-MRI approach has detected PD effects on SNc volume in two separate cohorts using two different MRI scanner models. With an NM-MRI contrast-based method to select the SNc region of interest (ROI), we have previously used the effective transverse relaxation rate R2^*^ to measure iron in SNc (47) and demonstrated that iron was significantly increased in SNc in PD (45). NM- and iron-sensitive MRI contrast regions in SN are largely non-overlapping in controls (48). The image processing method to select SNc ROI was based on NM-MRI contrast, which colocalizes with SNc-pigmented dopamine neurons (49). In this trial, we will use these imaging biomarkers to chart neurodegeneration in PD patients undergoing a previously tested intervention, with demonstrated clinical efficacy. We will evaluate a unique form of exercise in the PAIRED trial, which will be the first to examine the neuroprotective impact of partnered dance.

**Figure 1:**
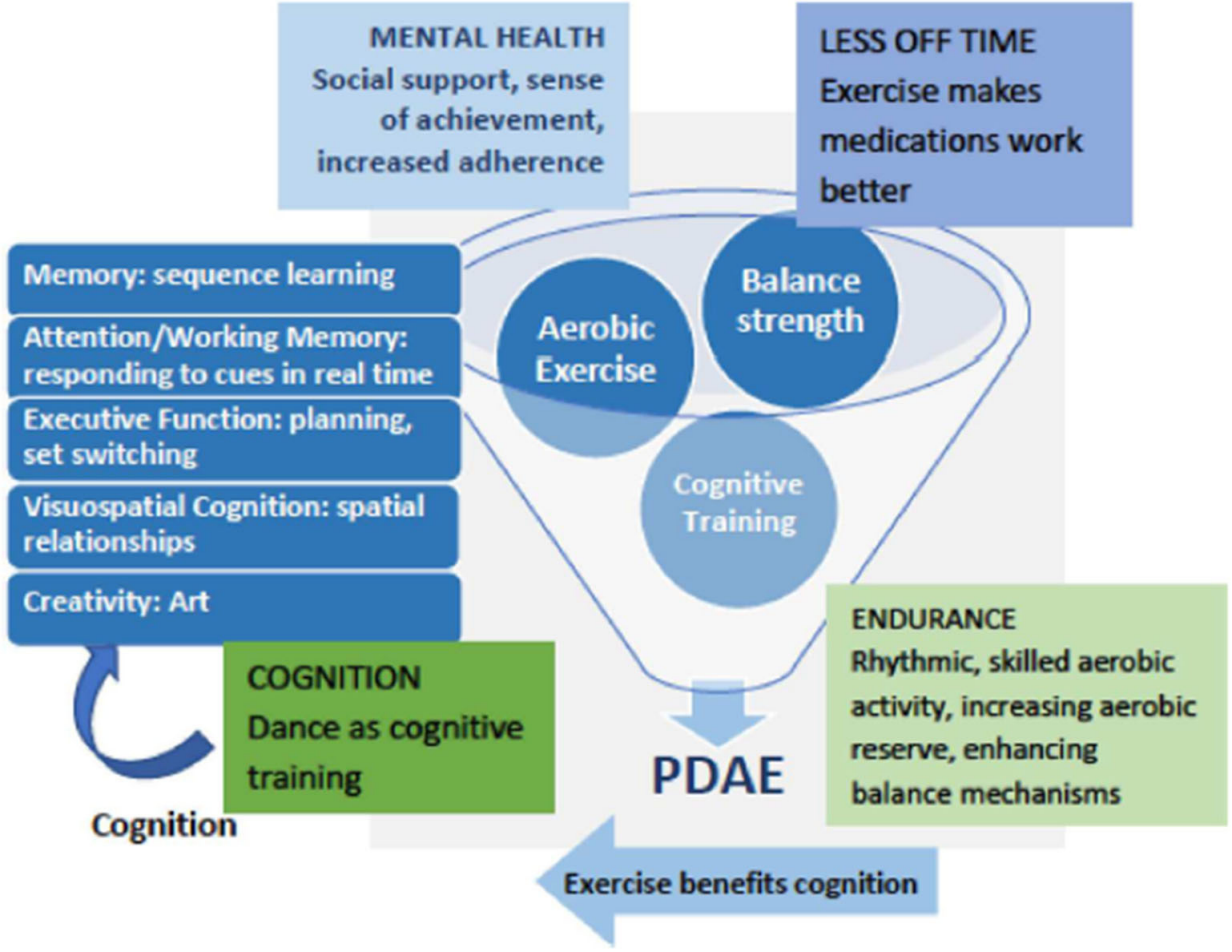
Hypothesized beneficial outcomes of Partnered Dance Aerobic Exercise.

Figure 1 depicts a conceptual framework for multidimensional benefits of partnered dance aerobic exercise (PDAE). A meta-analysis of randomized, controlled partnered dance trials in PD demonstrated improved symptom severity and balance (50). PDAE has been shown to improve motor function measured by motor symptoms, balance, preferred, backward and fast gait, mobility, endurance, and QOL (51–56). PDAE shares qualities of effective rehabilitation programs for people with PD, including repetition, intensity, task progression skill building, dynamic balance, and continual adjustments to the environment. To facilitate motor learning and make PDAE more accessible, the instructor deconstructs complex movements into simpler elements. PDAE movements are synchronized with musical rhythms and a partner, both of which are external cues that facilitate movement (57). PDAE is a ballroom dance and thus an AE that expends at least three metabolic equivalents (METs) (58). This amount is similar to usual walking in older adults (59), a low–moderate AE. PDAE requires cognitive engagement on multiple levels across multiple domains and has improved visuospatial cognition (56). After PDAE, our pilot data showed that patients reporting OFF-time at baseline reported reduced MRMF.

We offer two premises for our expected outcomes of our trial. Our premise for expected reductions in OFF-time post-Training and post-Maintenance phases of 16 months of PDAE is as follows: In our recently completed trial, we found that PDAE potentially reduces OFF-time in veterans with PD who reported MRMF. We randomly assigned 36 individuals with PD who reported experiencing OFF-time (11 female; age, 67.6 ± 7 years; UPDRS-III = 35.9 ± 11; PD duration = 7 ± 5 years) to 30 h PDAE or contact control (Education) over 3 months. PDAE participants improved more on the MDS-UPDRS-IV (which measures MRMF and OFF-time) (Table 1). However, it is important to compare PDAE's effect on OFF-time with the effect that another exercise program, e.g., walking, has on OFF-time. Uc et al. (30) reported an effect of *d* = 0.15 on OFF-time in 49 completers with PD of 6 months of community walking, meaning that they reported slightly less OFF-time (30). Comparing the effect of PDAE to walking in the Uc study on OFF-time, *d* = 0.89, in favor of PDAE.

**Table 1:** Comparison of change scores and differential effect sizes (Cohen's *d*) between 12 weeks of PDAE vs. time-matched education contact control **(A)** (EDU) and **(B)** WALK in mild–moderate PD.

		**Change scores**		***d*, PDAE**
**A. Outcome**	**Domain**	**PDAE**	**EDU**	**vs. EDU**
**Primary study outcomes**				
MDS UPDRS-IV	Motor complications	**2.23 (2.4)**	−0.3 (1.9)	1.09
Corsi product score	Spatial cognition	**6.9 (11.0)**	−3.4 (16.0)	0.76
**B. Outcome**	**Domain**	**PDAE**	WALK^*^	**d, PDAE vs. WALK**
OFF-time (30)	Motor complications	**2.23 (2.4)**	0.4 (1.7)	0.89

* *Data from (30); change scores >0 indicate improvement. Bold numbers indicate which group improved most*.

We explain the premise for expected improved spatial cognition and correlating measurable changes in the CNS. In our published study (56), 23 PDAE participants improved significantly on disease severity (*p* = 0.008; $\eta_{\text{p}}^{2}$ = 0.148) and spatial cognition (*p* = 0.021; $\eta_{\text{p}}^{2}$ = 0.121) compared to eight Education participants. PDAE participants also improved in executive function (*p* = 0.012; $\eta_{\text{p}}^{2}$ = 0.133). Gains were maintained 3 months postintervention. Table 1 reveals additional data from our lab comparing a contact control group (Education) vs. 18 randomly assigned participants who completed 30 h of PDAE for 3 months. Analyses of measures from the cognitive spatial domain using Corsi blocks assessment at pre- and post-20 lessons reveal a large effect between change scores of the Education group and the PDAE group (*d* = 0.76; Table 1).

**Figure 2:**
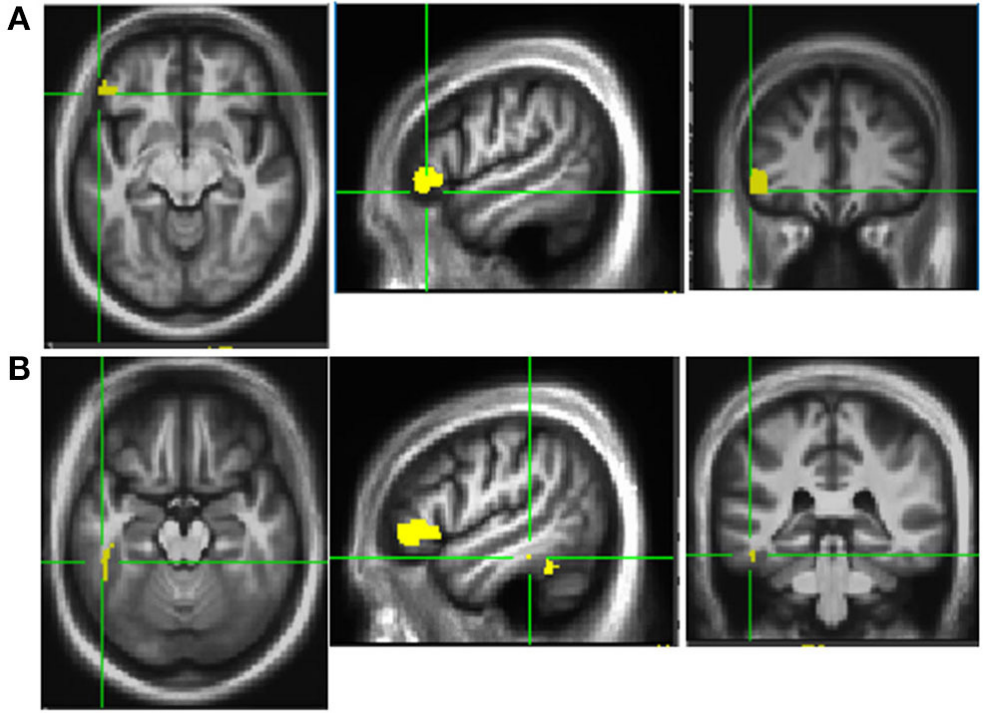
Activation patterns Post-PDAE. Increased activation was noted in left inferior frontal gyrus area 45 **(A)** and inferior temporal gyrus area 20 **(B)** at post compared to baseline after 12 weeks of PDAE.

This trial also examined neural mechanisms of dance-like movements for improving mobility and cognition. Before and after PDAE, participants laid in a scanner and performed a foot tapping task with the more affected limb in response to taps on their third ipsilateral metacarpal. Functional MRI (fMRI) data (atlas-based approach, *p* < 0.05) presented here are from 18 PDAE participants [7 female; age, 66.1 ± 9 years; PD duration, 6 ± 5 years; UPDRS-III, 34 ± 10; Montreal Cognitive Assessment (MoCA), 25.9 ± 3]. Posttraining, fMRI data revealed more activation in the left inferior frontal gyrus (area 45) [IFG (Figure 2A)] and the left inferior temporal gyrus (area 20) (ITG, Figure 2B). Post–pre changes in betas for each ROI correlate to improved spatial cognition in the same participants (*r* = 0.47 and *r* = 0.23). Other studies corroborate these findings: Modulation of the excitability of the IFG via rTMS was associated with enhanced cognitive processing in PD patients (60). Gray matter in the ITG increased in PD patients after balance training (61). These findings provide neural underpinnings for clinical findings, advancing understanding of pathologies and response to treatment and refining treatment approaches.

Our central hypothesis is that PDAE is a cognitively and socially engaging, potentially neuroprotective AE, which reduces OFF-time and improves spatial cognition in PD superior to walking aerobic exercise (WALK). Our goals are to identify whether 16 months of PDAE training (partnered, complex, skilled multirhythmic, and cognitively engaging walking activities) is superior to a WALK program for reducing OFF-time, improving cognition, and slowing neurodegeneration in PD. The WALK control group controls for AE and basic walking practice to isolate the cognitive aspects required to perform skills learned in PDAE.

Here, we describe the design and rationale of a clinical trial to determine whether PDAE is more effective than WALK at addressing OFF-time in people with mild–moderate PD. The PAIRED trial will (1) investigate PDAE's relationship with OFF-time and patient-reported drug efficacy, a patient-centered outcome that has received little attention in the context of exercise studies, over short (3 months) and long terms (13 month); (2) determine whether PDAE is a robust model for simultaneously delivering cognitive, motor skill, and AE training; (3) use novel imaging techniques to evaluate rates of neurodegeneration in the SNc of PD patients after long-term exercise intervention; and (4) investigate the efficacy of a novel, long-term 13-month maintenance phase of PDAE, which may have great utility for patients if less intense maintenance periods are shown to be effective after an initial more intensive training bout. We hypothesize that PDAE will improve OFF-time superior to WALK at a 3- and 16-month follow-up, that PDAE will improve spatial cognition superior to WALK, at 3- and at 16-month follow-up, and that we will note slowed SNc NM loss and decreased iron accumulation rate in PDAE vs. WALK.

## Study Design

### Overall Study Design

The PAIRED trial builds upon more than 10 years of research investigating unique motor, cognitive, and symptomatic benefits of PDAE. The study will perform a two arm, randomized controlled trial comparing efficacy of PDAE vs. WALK for improving OFF-time and visuospatial cognition among individuals with PD who report OFF-time. We will randomize *n* = 82 participants equally to 16 months of PDAE (*n* = 41) or to walking as aerobic exercise WALK (*n* = 41) in blocks of two to reduce variability within treatment conditions. Participants will be stratified by sex and Hoehn & Yahr (H&Y) staging (I, II, or III). The 16-month intervention period will consist of Training (3 months) and Maintenance (13 months) phases. In Training, participants will complete 20 biweekly 90-min sessions. In Maintenance, participants will complete 90-min weekly sessions. To evaluate SNc neurodegeneration over the observation period, we will image patients at study entry and 16-month follow-up using fMRI sequences. We will compare SNc volume and iron accumulation estimates in PDAE vs. WALK to assess whether PDAE is more neuroprotective.

Participants will be assessed at baseline, 3 months (immediately post-Training), and at 16 months (immediately post-Maintenance) for OFF-times, cognition, and neuroprotection, as evaluated in the SNc. At a standardized time of day, participants will be tested on all outcome measures at least 12 h after their last dose of antiparkinsonian medications. Furthermore, participants will keep an OFF-time diary to corroborate MDS-UPDRS-IV assessments. Participants will be ON medications during intervention. In addition to the primary motor and cognitive outcome measures, participants will be tested on secondary behavioral motor and cardiovascular and cognitive outcomes. Participants will undergo NM-MRI and fMRI scans at baseline and 3 and 16 months assessments to investigate mechanisms by which cognitive benefits have been obtained and replicated by PDAE and whether PDAE vs. WALK is neuroprotective, as exhibited by slowed rate of NM loss and iron accumulation and increased functional connectivity with spatial cognitive areas.

The proposed study has three aims. Aim 1 will evaluate whether exercise reduces OFF-time in PD patients who regularly suffer OFF-time. Aim 2 will examine the effects of a combined motor/cognitive intervention on cognition, specifically spatial cognition. In Aim 3, with physiological biomarkers, we aim to show that exercise slows neurodegeneration in humans with PD.

Primary outcome variables will be MDS-UPDRS-IV total score (Aim 1), Corsi Blocks product score (Aim 2), and rate of iron accumulation (Aim 3). Secondary outcome variables include motor, cognitive, and neuroanatomical variables. Motor variables include the distance walked during the 6-min walk test and a VO_2_ max measurement (Aim 2) and gait speed; cognitive variables include measures of executive function, attention, and additional visuospatial function measures (Aim 2). Neuroanatomical biomarker variables include the rate of loss of Snc NM volume (Aim 3).

### Power and Sample Size for the Primary Outcome

The trial is powered for the primary outcome, MDS UPDRS-IV total score. A differential effect size of *d* = 0.89 in the change of OFF-time for PDAE vs. walking using data collected by Uc et al. (30) was observed (Table 1) (30).

Given the study design's 3- and 16-month timepoints, we will use Holm's procedure (62) to control for multiple testing. The alpha levels will be 0.025 and 0.05 for *p*-values in ascending order for comparing PDAE and WALK in the changes from the baseline at 3 and 16 months. The observed effect size (*d* = 0.89) is from participants that finished their programs in the shorter duration pilot studies; therefore, we expect the intent-to-treat effect size will be reduced. Thus, we power the study for an attenuated effect of the observed effect: *d* = 0.7, which is 64% of the observed effect size in our pilot study or 79% of the estimated effect vs. walking, giving us 81 and 88% power for the comparisons at alpha level of 0.025 and 0.05, respectively. With a sample size of *n* = 41 completers per group, total *n* = 82, we additionally expect to have medium to high (60–80%) power to detect differences between the PDAE and WALK groups on the visuospatial outcome (Aim 2), given that the estimated effect size for Corsi Blocks is 0.76 vs. a cognitively engaging contact control (63). We will assume 25% attrition and will therefore recruit 102 participants with PD. For the exploratory variables, we will only focus on effect estimation instead of inference testing given that we do not know the effect size.

## Participants

Informed consent will be obtained prior to randomization. To account for 25% attrition, we will recruit 102 participants with PD for this study. For group assignment, we will recruit PD patients (age ≥40, Hoehn & Yahr stage I–III) who report OFF-time, indicated by score ≥1 on UPDRS-IV item 4.3, i.e., time spent in the OFF-state. Their PD diagnosis (ICD-10 G20^*^) will be based on established criteria (64) and determined by a board-certified neurologist with training in movement disorders. Individuals must present asymmetric symptoms that include at least three of the cardinal signs of PD (rigidity, bradykinesia, tremor, postural instability) and must show clear symptomatic benefit from antiparkinsonian medications (65). Participants will be recruited from the Atlanta VA HCS Movement Disorders clinic and the Emory Movement Disorders clinic, as well as at local Parkinson-related support groups, educational meetings, and community events. We will also recruit via the Michael J. Fox Foundation FoxFinder web-based registry. Based on the ≈60% prevalence of reported OFF-times among participants in ongoing studies and our ability to recruit one in five contacted participants, we plan to initially contact 656 potential participants to reach the enrollment goal.

## Intervention

Fidelity will be monitored by the investigators via weekly reports from interventionists and monthly class visits of both investigators. Dose for intervention will be measured by the time spent in the class, e.g., one class is a dose of 90 min. Volume of the intervention is defined as frequency times duration, and therefore, the volume will be ~180 min/week for the Training phase and 90 min/week for the Maintenance phase. The intensity of exercise will be measured with heart rate monitors.

### Intervention Dose Determinations

Participants will have two phases of intervention. In the 3-month Training phase, participants will be assigned to 12 weeks of 20, biweekly (90-min) lessons. Participants will attend lessons during the maintenance phase at least 3×/month.

Fall incidence has been extremely rare over 10 years, but fall prevention is a critical concern. All assistants for both PDAE and WALK will be trained on PD-related posture problems, monitoring balance, and anticipating and preventing falls with a 2-h experiential workshop.

### Partnered Dance Aerobic Exercise

PDAE has been shown to improve motor function as measured by motor symptoms, balance, preferred, backward and fast gait, mobility, endurance, and QOL (51–56). It is an adapted form of Argentine tango, aka Adapted tango. People with PD partner an individual without PD, e.g., a trained caregiver, friend, or university student. The instructor and several trained assistants will carefully monitor all participants. Class sizes will consist of 10 or fewer pairs of participants with PD and partners to maximize safety. Participants with PD will dance the follower role only and will dance with new partners (individuals without PD) every 15 min, a widely practiced method considered by the dance teaching community to enhance learning. Participants will engage in partnering exercises on how to interpret motor goals through touch, exercises to develop understanding of temporal relationship of movement to music, novel step introduction, and connecting previously learned and novel step elements. Frequent repetition and musical stereotypes may foster implicit learning or muscle memory (i.e., motor learning or procedural memory that involves consolidating a specific motor task into memory through repetition). Participants will not be required to memorize specific step patterns but will learn new steps in each class.

### Walking Aerobic Exercise

Participants in WALK will receive equivalent dose, volume, frequency, intensity, and duration of exercise to the PDAE group. They will receive equal contact and monitoring from study staff. WALK participants will report to the same facility and interact with the same interventionist and assistants. They will participate in sessions focused on at least 60 min of walking with breaks *ad libitum* and half hour balance and stretching. We have a designated safe and non-cluttered area for walking. WALK will also take place in groups, with research volunteers and assistants to ensure that PDAE and WALK participants both receive a socially engaging intervention.

## Pretest and Posttest Procedure Descriptions

Participants will be characterized for general health with a questionnaire at the first assessment. For blinded ratings, procedures will be videotaped and rated by research assistants. Through all assessments, to avoid fatigue, participants will have breaks *ad libitum* and will be offered snacks and water. The MDS-UPDRS parts I–IV will be administered to participants at all evaluations (1-week pretest, 1-week posttest, and 13-month posttest), by a qualified, MDS-certified and blinded rater. In addition to having reported OFF-time, participants must have a score >17 on the MoCA to screen out overt dementia (66). We will screen for depression with the Beck Depression Inventory-II (BDI-II), recommended for individuals with PD, with a cutoff of 18 (67). If participants are excluded for depression but receive treatment, they may be rescreened and considered for recruitment.

## Evaluations

### Self-Administered Psychosocial Questionnaires

To measure psychosocial impact of the social/touch human interactions inherent to partnered dance exercise, participants will have a week before each visit to complete questionnaires, i.e., Beck Depression Index II, the Composite Physical Function index. (68), the Physical Activity Scale for the Elderly, the PD Questionnaire-39 [patients have previously improved on this test as a result of PDAE (52)], and Short Form 12 Health Survey. We will also administer the five-item Satisfaction with Life Scale, which is valid in people with PD (69). As an exploratory measure, we will administer the Multidimensional Scale of Perceived Social Support (MSPSS) (70). The MSPSS aims to determine how social support factors are perceived by individuals, with three subscales to evaluate support by family, friends, and significant others. We will administer the Exit (Satisfaction) Questionnaire at 3- and 16-month assessments to survey satisfaction with Training and Maintenance phases of PDAE and WALK interventions and to elicit recommendations.

### Disease Severity

A blinded MDS-certified rater will administer the MDS-UPDRS parts I–IV at all evaluations. We will videotape Motor Subscale III. The primary outcome, the MDS-UPDRS-IV score (71), measures MRMF including dyskinesias, OFF-time, functional impact and complexity of fluctuations, and dystonia. We will administer a monthly OFF-state diary (72) for corroboration.

### Neuropsychological Assessment

Neuropsychological assessments will include the reverse Corsi blocks (73), a test of spatial function. The Brooks spatial memory and the Benton's judgment of line orientation will be administered as secondary, corroborative outcomes. We expect the interventions to benefit other aspects of cognition: attention/working memory, executive function, and visuospatial functions. Spatial cognition, attention, and executive function deficits result in impaired mobility, dual tasking problems, postural-gait disturbances, and ultimately falls (17, 18). The Trail Making Test and the Tower of London, both tests of executive function, will also be administered. Standard scores of simple conditions (using the timed motor responses) will take pure motor slowing into account. We will use different test versions at each time point to address practice effects. Analysis of the neuropsychological assessment data will be performed by a blinded analyst.

### Motor/Cardiovascular Assessments

#### Endurance: Six-Minute Walk Test

PDAE-mediated improvements in PD patients in 6-Min Walk Test (6MWT) performance have been demonstrated previously (53).

#### Gait Objective

Spatiotemporal parameters including velocity, stride length, stance percent, velocity variability, and other variables of preferred, backward, and fast-as-possible walking will be assessed by a 6-m computerized GAITRite walkway (CIR Systems, Inc. Havertown, PA, USA). We are examining backward walking because it is frequently practiced in PDAE and is more likely to be impaired earlier in the disease than forward walking and is more sensitive to change over time (74). We are examining fast as possible walking because it is sensitive to the effects of PDAE (53).

#### Balance

The Mini BEST test (75) and dynamic gait index (76).

#### Mobility and Dual Tasking

Single and Dual Timed Up and Go test (TUG) and four-square step test (77).

#### Cardiovascular Fitness Assessment

Participants will perform a submaximal exercise test on a cycle ergometer at baseline and 3- and 16-month assessments, per Young Men's Christian Association's (YMCA) submaximal test protocol. This submaximal test can estimate the participant's VO_2_ max and initial fitness level. This test uses a method in which heart rate (HR) workload values are obtained at 2–4 points and extrapolated to predict workload at the estimated maximum HR (e.g., 220-age). VO_2_ max is then calculated from the predicted maximum workload. A blinded assessor will ask participants to ride a stationary bicycle for four, 3-min stages. The first stage will be a warm-up at 50 revolutions per minute (rpm) at a power level of 25 W. We will continuously monitor HR and will not exceed 85% of age-predicted maximum HR. We will plot the average HR during the final 30 s of the second and third minutes against the workload. We will choose 3-min trial workloads based on participants' HR at the end of the warm-up. The fourth 3-min stage is the cooling down at the end of the test. A screening protocol based on a cardiovascular reserve test is more suitable for screening older adults (78).

#### Scanning/Neuroimaging

Participants will return for scanning within 3–4 days of clinical measures testing. We will examine participants in the OFF-state.

#### MRI Approach: Pulse Sequences and Standard Image Processing

NM-MRI, R2^*^, T_1_ MPRAGE, and T2^*^-weighted blood-oxygen-level-dependent (BOLD) fMRI data will be acquired using a Siemens Prisma-Fit 3T MRI scanner with a 64-channel receive-only head coil in a protocol with total scan time of 1 h. The NM-MRI sequence is the custom protocol developed by our group (41, 44). We will acquire NM-MRI data using a 2-D gradient echo sequence with a reduced flip-angle magnetization transfer preparation pulse (300°, 1.2 kHz off-resonance, 10 ms duration), TE/TR = 3.10/354, 15 contiguous slices, 416 × 512 imaging matrix, voxel size 0.39 × 0.39 × 3 mm^3^, seven measurements, flip angle = 40°, 470 Hz/pixel receiver bandwidth, and scan time of 17 min and 12 s (79). A blinded MRI analyst will carry out image processing to determine SNc volume using an automated approach previously shown to have high scan–rescan reproducibility (41, 79) and acquire R2^*^ data using conventional multiecho gradient echo sequence with four echoes spaced equally (see citation for parameters) (80). We will measure R2^*^ in the NM-MRI-defined SNc using a published image processing and analysis method (44, 45). We will use NM-MRI to delineate SNc and T2-weighted (iron-sensitive) MRI to delineate SNr. We will use an NM-MRI SNc population mask and a T2-weighted SNr population mask using data from a group of healthy controls (45) to select SNc and SNr ROIs with no operator-dependent steps (45).

#### Task-Based Functional MRI: Visuospatial Corsi Blocks: Block Span Task

Participants will engage in a scanner-adapted visuospatial task programmed in EPrime, using a modification of the block span task (BS) [as per (81)] in the scanner.

#### Task Description

Instead of using blocks, the scanner will visually present 10 squares. Each square will correspond to 1 of 10 keys (one for each finger) on the response pad. The experiment will contain 24 visual sequence periods. A sequence consists of the presentation of *n* randomized stimuli. The stimuli will be a single yellow block presented for 2s. We will customize the sequence length by adding one stimulus to baseline performance (*n* = BS + 1) collected outside the scanner before scanning. We will instruct participants to remember the location of the yellow squares as they appear, with no button press during this visual sequence period. At visual sequence period end, the participants will recall the sequence by pressing fingers on the response pad in the same order as the previously presented squares. The recall phase lasts 11s. Between two visual sequences, we will present an interstimulus interval (ISI) with jittered duration between 1.5 and 3.5s. Participants will perform the BS until they have two recall failures at the same span length. Before scanning, all patients will learn the pairing of each visual stimulus with the appropriate button response, using the less affected hand, and will practice until they are able to quickly and accurately match each stimulus with the appropriate response. We consider one sequence as correct when at least 70% of the spatial stimuli are recalled correctly to calculate patient correct response rate.

### Analyses

#### Aim 1

We will analyze the primary study outcome with a mixed effect model (treating subject as random) with between-subjects effect of group (PDAE vs. WALK) and within-subjects timepoints (study entry, 3-month follow-up, 16-month follow-up) plus their interaction term. If the interaction term is significant, we will test differences between PDAE and WALK in the change from baseline to 3 months and from baseline to 16 months using Holm's procedure to control for multiple testing. We will consider covariates sex and H&Y staging (<III, ≥III) in the analyses.

#### Aim 2

The secondary outcome Corsi Blocks Score will be analyzed using a similar mixed effects model.

#### Aim 3

We will use similar analyses. We will use mixed effects model except that it will be a two-group (PDAE vs. WALK) and a two-level variable for time (study entry vs. 16-month follow-up).

### Treatment of Non-compliance and Missing Data

We will follow enrolled patients until study end. We will analyze study outcomes according to original randomization scheme in an intent-to-treat approach. In case of missing data due to irreconcilable loss to follow-up, we will perform primary analyses using all available data with a regression-based multiple imputation procedure implemented in PROC MIXED and PROC MI in SAS software as per the SAS Institute (82). To evaluate sensitivity to systematic patterns of missingness, we will iterate multiple imputations by shifting or scaling the imputed values based on dropout models and comparing with estimated parameters. We will regard models to be “sensitive” if imputed values alter parameters by ≥15%.

## Discussion

Most people with PD experience OFF-time that severely impacts QOL (83–85). Little research has explicitly looked at the potentially synergistic relationship between the patient experience of drug efficacy and exercise therapies. We will provide insight into the beneficial outcomes of treatments like PDAE, in concert with best practice pharmacological treatment. Based on promising data, this trial will provide longitudinal short- and long-term effects of an evidence-based training to inform clinicians of the relationship between effective treatment and their pharmacological regimens. The patient experience of drug efficacy is multifactorial, involving confluence of biochemical pathway processes, motor and cognitive status, and social situations (14, 86–91). If our prediction is correct, reduced OFF-time could result from enhanced aerobic fitness, psychosocial engagement, or motor–cognitive skill enhancement via the therapy. Exercise may allow participants treated with levodopa to use dopamine more efficiently and improve motor symptoms (54, 88, 92). These beneficial effects may be reflected in better patient experience of drug efficacy. Recent evidence supports that AE involving motor skills differentially affects frontal–striatal-related circuits more than pure AE (22).

Combined physical/cognitive trainings done either simultaneously or in a tight series, e.g., stationary bicycling while completing computerized cognitive training on a screen, may benefit motor and cognitive function in humans, based on animal literature and some well-designed human studies (93–95). Spatial memory has been shown to improve with aerobic and resistance training exercises (96). Our own research has shown that Adapted Tango improves disease severity, balance, spatial cognition, and executive function (56). OFF-time is directly related to dopamine replacement therapy. Although the relationship between aerobic exercise and spatial cognitive function among PD patients with OFF-time is unclear, since exercise attenuates the levodopa-induced medication-related motor complications (88), improves incidental sequence learning (97), and improves visual memory and visuospatial abilities (98), it stands to reason that the exercise-dependent improvement in OFF-time may further improve spatial cognitive function.

In PDAE, physical and cognitive activities are solidly and seamlessly integrated. This project will reveal whether PDAE is efficient for spatial cognitive rehabilitation. If so, an advantage of PDAE is that it is more engaging and enjoyable than an alternative, e.g., computerized cognitive training done with exercise. We hypothesize that PDAE will rehabilitate spatial cognition in PD. We explain our reasoning here. During PDAE, one can learn coordinated relationships between people, places, and objects and one performs spatial navigation with a partner and other individuals. PDAE also requires strong cognitive engagement in several domains: attention to partner; storing new steps in memory and recalling previously learned steps; detecting and interpreting musical beats; coordinating body movement with external musical source and partner; and using working memory via learning steps and practicing them and language, via a new vocabulary for a dance context stringing together steps into phrases, and holding “conversations” with partner, via non-verbal communication. We expect positive effects of the AE component of PDAE on symptoms (30); however, the WALK group controls for aerobic exercise to isolate the cognitive elements inherent to PDAE. Correlations with improved non-trained spatial tasks suggest transfer effects of PDAE (99). Cognitive training for people with PD has induced long-term effects in brain activation (18 months post) (100). The cognitive elements of PDAE would rehabilitate cognition similarly over the long term. Furthermore, cardiovascular fitness modulates brain activation associated with spatial learning (101). We plan to uncover neural mechanisms of benefit to cognitive domains via fMRI.

We propose that the social support/human touch and cognitive engagement inherent to PDAE will benefit participants more in OFF-time and in cognitive function. It has not been unequivocally demonstrated that any form of exercise can slow neurodegeneration in humans although aerobic exercise has been demonstrated to slow neurodegeneration in animal models (86). As such, we propose using PDAE as a model in humans, given PDAE's replicated beneficial effects in multiple studies (53, 92, 102). Exercise has been touted as neuroprotective in humans based on findings in rodent studies; however, evidence is needed within humans to firmly establish exercise as neuroprotective. PDAE evidently benefits motor and cognitive function and may improve drug efficacy; with better understanding of neurodegenerative processes after the course of long-term intervention, novel, and more effective therapies can be developed. A potential neuroprotective effect of PDAE might be reflected in reduced iron accumulation and slowed NM loss. Furthermore, the evidence of a correlation between cognitive benefits and neural activity in brain regions anatomically referable to these cognitive functions suggests a relationship between slowed neurodegeneration and the cognitive impact of PDAE, which might exceed that of WALK. Given the previous benefits seen with exercise modalities, and specifically with PDAE, it is reasonable to expect some impact upon the neurodegenerative process, which can be robustly and rigorously characterized with our biomarkers.

The therapeutic benefits of dance have been studied since the 1900s and have produced interesting findings; however, additional clinical trials are necessary to strengthen the evidence in support of partnered dance treatment for people with PD.

## Data Availability Statement

The raw data supporting the conclusions of this article will be made available by the authors, without reservation, upon request.

## Ethics Statement

This study has been approved by both the Emory IRB and the VA Research and Development committees. Written informed consent will be obtained from all participants prior to participation.

## Author Contributions

MH, BC, ME, DH, JN, JL, XC, JM, and VK contributed to conception and design of the study. AB and JJ wrote the first draft of the manuscript, MH extensively edited the manuscript. All authors contributed to manuscript revision and read and approved the submitted version.

## Conflict of Interest

The authors declare that the research was conducted in the absence of any commercial or financial relationships that could be construed as a potential conflict of interest.

## References

[1] Kowal SL Dall TM Chakrabarti R Storm MV Jain A. The current and projected economic burden of Parkinson's disease in the United States. Mov Disord. (2013) 28:311–8. 10.1002/mds.25292 23436720

[2] Tsugawa J Onozawa R Fukae J Mishima T Fujioka S Tsuboi Y. Impact of insufficient drug efficacy of antiparkinson agents on patient's quality of life: a cross-sectional study. BMC Neurol. (2015) 15:105. 10.1186/s12883-015-0360-y 26143184 PMC4491278

[3] Marras C Armstrong MJ Meaney CA Fox S Rothberg B Reginold W. Measuring mild cognitive impairment in patients with Parkinson's disease. Mov Disord. (2013) 28:626–33. 10.1002/mds.25426 23520128 PMC4524474

[4] Marsden CD Parkes JD. On-off effects in patients with Parkinson's disease on chronic levodopa therapy. Lancet. (1976) 1:292–6. 10.1016/S0140-6736(76)91416-1 55599

[5] Blindauer K Shoulson I Oakes D Kieburtz K Schwid S Fahn S. A randomized controlled trial of etilevodopa in patients with Parkinson disease who have motor fluctuations. Arch Neurol. (2006) 63:210–6. 10.1001/archneur.63.2.210 16476809

[6] Denny AP Behari M. Motor fluctuations in Parkinson's disease. J Neurol Sci. (1999) 165:18–23. 10.1016/S0022-510X(99)00052-0 10426141

[7] Ahlskog JE Muenter MD. Frequency of levodopa-related dyskinesias and motor fluctuations as estimated from the cumulative literature. Mov Disord. (2001) 16:448–58. 10.1002/mds.1090 11391738

[8] Frazzitta G Bertotti G Morelli M Riboldazzi G Pelosin E Balbi P. Rehabilitation improves dyskinesias in Parkinsonian patients: a pilot study comparing two different rehabilitative treatments. NeuroRehabilitation. (2012) 30:295–301. 10.3233/NRE-2012-0758 22672943

[9] Frazzitta G Bertotti G Riboldazzi G Turla M Uccellini D Boveri N. Effectiveness of intensive inpatient rehabilitation treatment on disease progression in parkinsonian patients: a randomized controlled trial with 1-year follow-up. Neurorehabil Neural Repair. (2012) 26:144–50. 10.1177/1545968311416990 21844282

[10] Jankovic J Aguilar LG. Current approaches to the treatment of Parkinson's disease. Neuropsychiatr Dis Treat. (2008) 4:743–57. 10.2147/NDT.S2006 19043519 PMC2536542

[11] Wullner U Fuchs G Reketat N Randerath O Kassubek J. Requirements for Parkinson's disease pharmacotherapy from the patients' perspective: a questionnaire-based survey. Curr Med Res Opin. (2012) 28:1239–46. 10.1185/03007995.2012.702101 22686959

[12] Cilia R Cereda E Klersy C Canesi M Zecchinelli AL Mariani CB. Parkinson's disease beyond 20 years. J Neurol Neurosurg Psychiatry. (2015) 86:849–55. 10.1136/jnnp-2014-308786 25280915

[13] Broeders M de Bie RM Velseboer DC Speelman JD Muslimovic D Schmand B. Evolution of mild cognitive impairment in Parkinson disease. Neurology. (2013) 81:346–52. 10.1212/WNL.0b013e31829c5c86 23794682

[14] Lundqvist C. Continuous levodopa for advanced Parkinson's disease. Neuropsychiatr Dis Treat. (2007) 3:335–48. 19300565 PMC2654791

[15] Possin KL. Visual spatial cognition in neurodegenerative disease. Neurocase. (2010) 16:466–87. 10.1080/13554791003730600 20526954 PMC3028935

[16] Klencklen G Despres O Dufour A. What do we know about aging and spatial cognition? Reviews and perspectives. Ageing Res Rev. (2012) 11:123–35. 10.1016/j.arr.2011.10.001 22085884

[17] Mak MK Wong A Pang MY. Impaired executive function can predict recurrent falls in Parkinson's disease. Arch Phys Med Rehabil. (2014) 95:2390–5. 10.1016/j.apmr.2014.08.006 25175162

[18] Paul SS Sherrington C Canning CG Fung VS Close JC Lord SR. The relative contribution of physical and cognitive fall risk factors in people with Parkinson's disease: a large prospective cohort study. Neurorehabil Neural Repair. (2014) 28:282–90. 10.1177/1545968313508470 24243915

[19] Blanchet PJ Brefel-Courbon C. Chronic pain and pain processing in Parkinson's disease. Prog Neuropsychopharmacol Biol Psychiatry. (2017) 87 (Pt. B):200–6. 10.1016/j.pnpbp.2017.10.010 29031913

[20] Reynolds GO Otto MW Ellis TD Cronin-Golomb A. The therapeutic potential of exercise to improve mood, cognition, and sleep in parkinson's disease. Mov Disord. (2016) 31:23–38. 10.1002/mds.26484 26715466 PMC4724300

[21] Ahlskog JE. Does vigorous exercise have a neuroprotective effect in Parkinson disease? Neurology. (2011) 77:288–94. 10.1212/WNL.0b013e318225ab66 21768599 PMC3136051

[22] Petzinger GM Holschneider DP Fisher BE McEwen S Kintz N Halliday M. The Effects of exercise on dopamine neurotransmission in parkinson's disease: targeting neuroplasticity to modulate basal ganglia circuitry. Brain Plast. (2015) 1:29–39. 10.3233/BPL-150021 26512345 PMC4621077

[23] Fisher BE Li Q Nacca A Salem GJ Song J Yip J. Treadmill exercise elevates striatal dopamine D2 receptor binding potential in patients with early Parkinson's disease. Neuroreport. (2013) 24:509–14. 10.1097/WNR.0b013e328361dc13 23636255

[24] Mouradian MM Juncos JL Serrati C Fabbrini G Palmeri S Chase TN. Exercise and the antiparkinsonian response to levodopa. Clin Neuropharmacol. (1987) 10:351–5. 10.1097/00002826-198708000-00005 3503678

[25] Carter JH Nutt JG Woodward WR. The effect of exercise on levodopa absorption. Neurology. (1992) 42:2042–5. 10.1212/WNL.42.10.2042 1407589

[26] Reuter I Harder S Engelhardt M Baas H. The effect of exercise on pharmacokinetics and pharmacodynamics of levodopa. Mov Disord. (2000) 15:862–8. 10.1002/1531-8257(200009)15:5<862::AID-MDS1015>3.0.CO;2-S 11009191

[27] Muhlack S Welnic J Woitalla D Muller T. Exercise improves efficacy of levodopa in patients with Parkinson's disease. Mov Disord. (2007) 22:427–30. 10.1002/mds.21346 17226855

[28] David FJ Robichaud JA Vaillancourt DE Poon C Kohrt WM Comella CL. Progressive resistance exercise restores some properties of the triphasic EMG pattern and improves bradykinesia: the PRET-PD randomized clinical trial. J Neurophysiol. (2016) 116:2298–311. 10.1152/jn.01067.2015 27582297 PMC5110637

[29] Dibble LE Foreman KB Addison O Marcus RL LaStayo PC. Exercise and medication effects on persons with Parkinson disease across the domains of disability: a randomized clinical trial. J Neurol Phys Ther. (2015) 39:85–92. 10.1097/NPT.0000000000000086 25742370 PMC4366306

[30] Uc EY Doerschug KC Magnotta V Dawson JD Thomsen TR Kline JN. Phase I/II randomized trial of aerobic exercise in Parkinson disease in a community setting. Neurology. (2014) 83:413–25. 10.1212/WNL.0000000000000644 24991037 PMC4132568

[31] Duchesne C Lungu O Nadeau A Robillard ME Bore A Bobeuf F. Enhancing both motor and cognitive functioning in Parkinson's disease: aerobic exercise as a rehabilitative intervention. Brain Cogn. (2015) 99:68–77. 10.1016/j.bandc.2015.07.005 26263381

[32] David FJ Robichaud JA Leurgans SE Poon C Kohrt WM Goldman JG. Exercise improves cognition in Parkinson's disease: the PRET-PD randomized, clinical trial. Mov Disord. (2015) 30:1657–63. 10.1002/mds.26291 26148003 PMC4609235

[33] Hotting K Holzschneider K Stenzel A Wolbers T Roder B. Effects of a cognitive training on spatial learning and associated functional brain activations. BMC Neurosci. (2013) 14:73. 10.1186/1471-2202-14-73 23870447 PMC3729599

[34] Kempermann G. The neurogenic reserve hypothesis: what is adult hippocampal neurogenesis good for? Trends Neurosci. (2008) 31:163–9. 10.1016/j.tins.2008.01.002 18329110

[35] Prakash RS Voss MW Erickson KI Kramer AF. Physical activity and cognitive vitality. Annu Rev Psychol. (2015) 66:769–97. 10.1146/annurev-psych-010814-015249 25251492

[36] Erickson KI Voss MW Prakash RS Basak C Szabo A Chaddock L. Exercise training increases size of hippocampus and improves memory. Proc Natl Acad Sci USA. (2011) 108:3017–22. 10.1073/pnas.1015950108 21282661 PMC3041121

[37] Hayes SM Salat DH Forman DE Sperling RA Verfaellie M. Cardiorespiratory fitness is associated with white matter integrity in aging. Ann Clin Transl Neurol. (2015) 2:688–98. 10.1002/acn3.204 26125043 PMC4479528

[38] Wang Z Myers KG Guo Y Ocampo MA Pang RD Jakowec MW. Functional reorganization of motor and limbic circuits after exercise training in a rat model of bilateral parkinsonism. PLoS ONE. (2013) 8:e80058. 10.1371/journal.pone.0080058 24278239 PMC3836982

[39] Fearnley JM Lees AJ. Ageing and Parkinson's disease: substantia nigra regional selectivity. Brain. (1991) 114 (Pt. 5):2283–301. 10.1093/brain/114.5.2283 1933245

[40] Sian-Hulsmann J Mandel S Youdim MB Riederer P. The relevance of iron in the pathogenesis of Parkinson's disease. J Neurochem. (2011) 118:939–57. 10.1111/j.1471-4159.2010.07132.x 21138437

[41] Chen X Huddleston DE Langley J Ahn S Barnum CJ Factor SA. Simultaneous imaging of locus coeruleus and substantia nigra with a quantitative neuromelanin MRI approach. Magn Reson Imaging. (2014) 32:1301–6. 10.1016/j.mri.2014.07.003 25086330

[42] Du G Lewis MM Sen S Wang J Shaffer ML Styner M. Imaging nigral pathology and clinical progression in Parkinson's disease. Mov Disord. (2012) 27:1636–43. 10.1002/mds.25182 23008179 PMC3510346

[43] Huddleston D Langley J McMurray R De Louis F Factor S Hu X Advanced neuromelanin sensitive MRI measures detect Parkinson's disease effects in catecholamine nuclei: discovery and validation in separate cohorts. Neurology. (2017). 88 (16 Suppl):P1. 076.

[44] Huddleston DE Langley J Sedlacik J Boelmans K Factor SA Hu XP. *In vivo* detection of lateral-ventral tier nigral degeneration in Parkinson's disease. Hum Brain Mapp. (2017) 38:2627–34. 10.1002/hbm.23547 28240402 PMC5385149

[45] Langley J Huddleston DE Merritt M Chen X McMurray R Silver M. Diffusion tensor imaging of the substantia nigra in Parkinson's disease revisited. Hum Brain Mapp. (2016) 37:2547–56. 10.1002/hbm.23192 27029026 PMC4905784

[46] Trujillo P Summers PE Ferrari E Zucca FA Sturini M Mainardi LT. Contrast mechanisms associated with neuromelanin-MRI. Magn Reson Med. (2016) 78:1790–800. 10.1002/mrm.26584 28019018

[47] Langkammer C Schweser F Krebs N Deistung A Goessler W Scheurer E. Quantitative susceptibility mapping (QSM) as a means to measure brain iron? A post mortem validation study. Neuroimage. (2012) 62:1593–9. 10.1016/j.neuroimage.2012.05.049 22634862 PMC3413885

[48] Langley J Huddleston DE Chen X Sedlacik J Zachariah N Hu X. A multicontrast approach for comprehensive imaging of substantia nigra. Neuroimage. (2015) 112:7–13. 10.1016/j.neuroimage.2015.02.045 25731994 PMC4415274

[49] Kitao S Matsusue E Fujii S Miyoshi F Kaminou T Kato S. Correlation between pathology and neuromelanin MR imaging in Parkinson's disease and dementia with lewy bodies. Neuroradiology. (2013) 55:947–53. 10.1007/s00234-013-1199-9 23673875

[50] Lotzke D Ostermann T Bussing A. Argentine tango in Parkinson disease–a systematic review and meta-analysis. BMC Neurol. (2015) 15:226. 10.1186/s12883-015-0484-0 26542475 PMC4636067

[51] Hackney ME Earhart GM. Effects of dance on movement control in Parkinson's disease: a comparison of argentine tango and American ballroom. J Rehabil Med. (2009) 41:475–81. 10.2340/16501977-0362 19479161 PMC2688709

[52] Hackney ME Earhart GM. Health-related quality of life and alternative forms of exercise in Parkinson disease. Parkinsonism Relat Disord. (2009) 15:644–8. 10.1016/j.parkreldis.2009.03.003 19329350 PMC2783812

[53] Hackney ME Earhart GM. Effects of dance on gait and balance in Parkinson's disease: a comparison of partnered and nonpartnered dance movement. Neurorehabil Neural Repair. (2010) 24:384–92. 10.1177/1545968309353329 20008820 PMC2900796

[54] Hackney ME Kantorovich S Levin R Earhart GM Effects of tango on functional mobility in Parkinson's disease: a preliminary study. J Neurol Phys Ther. (2007) 31:173–9. 10.1097/NPT.0b013e31815ce78b 18172414

[55] McKay JL Ting LH Hackney ME. Balance, body motion, and muscle activity after high-volume short-term dance-based rehabilitation in persons with Parkinson disease: a pilot study. J Neurol Phys Ther. (2016) 40:257–68. 10.1097/NPT.0000000000000150 27576092 PMC5025380

[56] McKee KE Hackney ME. The effects of adapted tango on spatial cognition and disease severity in Parkinson's disease. J Mot Behav. (2013) 45:519–29. 10.1080/00222895.2013.834288 24116748 PMC3864026

[57] Howe TE Lovgreen B Cody FW Ashton VJ Oldham JA. Auditory cues can modify the gait of persons with early-stage Parkinson's disease: a method for enhancing parkinsonian walking performance? Clin Rehabil. (2003) 17:363–7. 10.1191/0269215503cr621oa 12785243

[58] Jette M Sidney K Blumchen G. Metabolic equivalents (METS) in exercise testing, exercise prescription, and evaluation of functional capacity. Clin Cardiol. (1990) 13:555–65. 10.1002/clc.4960130809 2204507

[59] Knaggs JD Larkin KA Manini TM. Metabolic cost of daily activities and effect of mobility impairment in older adults. J Am Geriatr Soc. (2011) 59:2118–23. 10.1111/j.1532-5415.2011.03655.x 22091979 PMC3874461

[60] Srovnalova H Marecek R Rektorova I. The role of the inferior frontal gyri in cognitive processing of patients with Parkinson's disease: a pilot rTMS study. Mov Disord. (2011) 26:1545–8. 10.1002/mds.23663 21480374

[61] Sehm B Taubert M Conde V Weise D Classen J Dukart J. Structural brain plasticity in Parkinson's disease induced by balance training. Neurobiol Aging. (2014) 35:232–9. 10.1016/j.neurobiolaging.2013.06.021 23916062

[62] Holm S A simple sequentially rejective multiple test procedure. Scand J Stat. (1979) 6:65–70. Retrieved from <Go to ISI>://WOS:A1979JY78700003

[63] Cohen J. A power primer. Psychol Bull. (1992) 112:155–9. 10.1037/0033-2909.112.1.155 19565683

[64] Hughes AJ Daniel SE Kilford L Lees AJ. Accuracy of clinical diagnosis of idiopathic Parkinson's disease: a clinico-pathological study of 100 cases. J Neurol Neurosurg Psychiatry. (1992) 55:181–4. 10.1136/jnnp.55.3.181 1564476 PMC1014720

[65] Kempster PA Williams DR Selikhova M Holton J Revesz T Lees AJ. Patterns of levodopa response in Parkinson's disease: a clinico-pathological study. Brain. (2007) 130 (Pt. 8):2123–8. 10.1093/brain/awm142 17586867

[66] Litvan I Goldman JG Troster AI Schmand BA Weintraub D Petersen RC. Diagnostic criteria for mild cognitive impairment in Parkinson's disease: movement disorder society task force guidelines. Mov Disord. (2012) 27:349–56. 10.1002/mds.24893 22275317 PMC3641655

[67] Schrag A Barone P Brown RG Leentjens AF McDonald WM Starkstein S. Depression rating scales in Parkinson's disease: critique and recommendations. Mov Disord. (2007) 22:1077–92. 10.1002/mds.21333 17394234 PMC2040268

[68] Rikli RE Jones CJ Evaluating functional fitness of older men and women in the field setting. Phys Fit Health Promot Act Aging. (2001) 17:11–20. Retrieved from <Go to ISI>://WOS:000172148000002

[69] Zimet GD Powell SS Farley GK Werkman S Berkoff KA. Psychometric characteristics of the multidimensional scale of perceived social support. J Pers Assess. (1990) 55:610–7. 10.1207/s15327752jpa5503andamp;4_17 2280326

[70] Lovereide L Hagell P. Measuring life satisfaction in parkinson's disease and healthy controls using the satisfaction with life scale. PLoS ONE. (2016) 11:e0163931. 10.1371/journal.pone.0163931 27776131 PMC5077103

[71] Goetz CG Tilley BC Shaftman SR Stebbins GT Fahn S Martinez-Martin P. Movement disorder society-sponsored revision of the unified Parkinson's disease rating scale (MDS-UPDRS): scale presentation and clinimetric testing results. Mov Disord. (2008) 23:2129–70. 10.1002/mds.22340 19025984

[72] Hauser RA Friedlander J Zesiewicz TA Adler CH Seeberger LC O'Brien CF. A home diary to assess functional status in patients with Parkinson's disease with motor fluctuations and dyskinesia. Clin Neuropharmacol. (2000) 23:75–81. 10.1097/00002826-200003000-00003 10803796

[73] Kessels RP van den Berg E Ruis C Brands AM. The backward span of the corsi block-tapping task and its association with the WAIS-III digit span. Assessment. (2008) 15:426–34. 10.1177/1073191108315611 18483192

[74] Hackney ME Earhart GM Backward walking in Parkinson's disease. Mov Disord. (2009) 24:218–23. 10.1002/mds.22330 18951535 PMC2945224

[75] King L Horak F. On the mini-BESTest: scoring and the reporting of total scores. Phys Ther. (2013) 93:571–5. 10.2522/ptj.2013.93.4.571 23547173

[76] Bloem BR Marinus J Almeida Q Dibble L Nieuwboer A Post B. Measurement instruments to assess posture, gait, and balance in Parkinson's disease: critique and recommendations. Mov Disord. (2016) 31:1342–55. 10.1002/mds.26572 26945525

[77] Dite W Temple VA. A clinical test of stepping and change of direction to identify multiple falling older adults. Arch Phys Med Rehabil. (2002) 83:1566–71. 10.1053/apmr.2002.35469 12422327

[78] Gill TM DiPietro L Krumholz HM. Role of exercise stress testing and safety monitoring for older persons starting an exercise program. JAMA. (2000) 284:342–9. 10.1001/jama.284.3.342 10891966

[79] Langley J Huddleston DE Liu CJ Hu X. Reproducibility of locus coeruleus and substantia nigra imaging with neuromelanin sensitive MRI. MAGMA. (2017) 30:121–5. 10.1007/s10334-016-0590-z 27687624

[80] Barbosa JH Santos AC Tumas V Liu M Zheng W Haacke EM. Quantifying brain iron deposition in patients with Parkinson's disease using quantitative susceptibility mapping, R2 and R2. Magn Reson Imaging. (2015) 33:559–65. 10.1016/j.mri.2015.02.021 25721997

[81] Doucet G Osipowicz K Sharan A Sperling MR Tracy JI. Hippocampal functional connectivity patterns during spatial working memory differ in right versus left temporal lobe epilepsy. Brain Connect. (2013) 3:398–406. 10.1089/brain.2013.0158 23705755 PMC3749700

[82] Yuan Y Multiple Imputation for Missing Data: Concepts and New Development. Rockville, MD: SAS Institute Inc (2005).

[83] Klietz M Tulke A Müschen LH Paracka L Schrader C Dressler DW. Impaired quality of life and need for palliative care in a German cohort of advanced Parkinson's disease patients. Front Neurol. (2018) 9:120. 10.3389/fneur.2018.00120 29559949 PMC5845640

[84] Rodríguez-Violante M Ospina-García N Dávila-Avila NM Cruz-Fino D Cruz-Landero A Cervantes-Arriaga A. Motor and non-motor wearing-off and its impact in the quality of life of patients with Parkinson's disease. Arquivos de Neuro-Psiquiatria. (2018) 76:517–21. 10.1590/0004-282x20180074 30231124

[85] Wu J Lim E-C Nadkarni NV Tan E-K Kumar PM. The impact of levodopa therapy-induced complications on quality of life in Parkinson's disease patients in Singapore. Sci Rep. (2019) 9:9248. 10.1038/s41598-019-45110-5 31239456 PMC6593098

[86] Ahlskog JE. Aerobic exercise: evidence for a direct brain effect to slow Parkinson disease progression. Mayo Clin Proc. (2018) 93:360–72. 10.1016/j.mayocp.2017.12.015 29502566

[87] Aguiar AS Lopes SC Tristão FSM Rial D de Oliveira G da Cunha C. Exercise improves cognitive impairment and dopamine metabolism in MPTP-treated mice. Neurotox Res. (2016) 29:118–25. 10.1007/s12640-015-9566-4 26464310

[88] Aguiar AS Moreira ELG Hoeller AA Oliveira PA Córdova FM Glaser V. Exercise attenuates levodopa-induced dyskinesia in 6-hydroxydopamine-lesioned mice. Neuroscience. (2013) 243:46–53. 10.1016/j.neuroscience.2013.03.039 23558088

[89] Babiloni C Del Percio C Lizio R Noce G Lopez S Soricelli A. Levodopa may affect cortical excitability in Parkinson's disease patients with cognitive deficits as revealed by reduced activity of cortical sources of resting state electroencephalographic rhythms. Neurobiol Aging. (2019) 73:9–20. 10.1016/j.neurobiolaging.2018.08.010 30312790

[90] Moguel-Cobos G Saldivar C Goslar PW Shill HA. The relationship between social anxiety disorder and motor symptoms of Parkinson disease: a pilot study. Psychosomatics. (2020) 61:321–6. 10.1016/j.psym.2020.03.006 32386770

[91] Xu X Fu Z Le W Chapter two - exercise and Parkinson's disease. In: Yau SY, So KF, editors. International Review of Neurobiology. Academic Press (2019). p. 45–74. 10.1016/bs.irn.2019.06.003 31607362

[92] Hackney ME Byers C Butler G Sweeney M Rossbach L Bozzorg A. Adapted tango improves mobility, motor-cognitive function, and gait but not cognition in older adults in independent living. J Am Geriatr Soc. (2015) 63:2105–13. 10.1111/jgs.13650 26456371

[93] de Bruin ED van Het Reve E Murer K. A randomized controlled pilot study assessing the feasibility of combined motor-cognitive training and its effect on gait characteristics in the elderly. Clin Rehabil. (2013) 27:215–25. 10.1177/0269215512453352 22865831

[94] Wiloth S Lemke N Werner C Hauer K. Validation of a computerized, game-based assessment strategy to measure training effects on motor-cognitive functions in people with dementia. JMIR Serious Games. (2016) 4:e12. 10.2196/games.5696 27432746 PMC4969551

[95] Wiloth S Werner C Lemke NC Bauer J Hauer K. Motor-cognitive effects of a computerized game-based training method in people with dementia: a randomized controlled trial. Aging Mental Health. (2018) 22:1130–41. 10.1080/13607863.2017.1348472 28682124

[96] Cassilhas RC Lee KS Fernandes J Oliveira MGM Tufik S Meeusen R Spatial memory is improved by aerobic and resistance exercise through divergent molecular mechanisms. Neuroscience. (2012) 202:309–17. 10.1016/j.neuroscience.2011.11.029 22155655

[97] Beigi M Wilkinson L Gobet F Parton A Jahanshahi M. Levodopa medication improves incidental sequence learning in Parkinson's disease. Neuropsychologia. (2016) 93:53–60. 10.1016/j.neuropsychologia.2016.09.019 27686948 PMC5155668

[98] Kulisevsky J García-Sánchez C Berthier ML Barbanoj M Pascual-Sedano B Gironell A. Chronic effects of dopaminergic replacement on cognitive function in Parkinson's disease: a two-year follow-up study of previously untreated patients. Mov Disord. (2000) 15:613–26. 10.1002/1531-8257(200007)15:4<613::AID-MDS1005>3.0.CO 10928571

[99] Heinzel S Lorenz RC Pelz P Heinz A Walter H Kathmann N. Neural correlates of training and transfer effects in working memory in older adults. Neuroimage. (2016) 134:236–49. 10.1016/j.neuroimage.2016.03.068 27046110

[100] Diez-Cirarda M Ojeda N Pena J Cabrera-Zubizarreta A Lucas-Jimenez O Gomez-Esteban JC. Long-term effects of cognitive rehabilitation on brain, functional outcome and cognition in Parkinson's disease. Eur J Neurol. (2017) 100:5–12. 10.1111/ene.13472 28940855 PMC5765471

[101] Holzschneider K Wolbers T Roder B Hotting K. Cardiovascular fitness modulates brain activation associated with spatial learning. Neuroimage. (2012) 59:3003–14. 10.1016/j.neuroimage.2011.10.021 22027496

[102] Hackney ME Hall CD Echt KV Wolf SL. Dancing for balance: feasibility and efficacy in oldest-old adults with visual impairment. Nurs Res. (2013) 62:138–43. 10.1097/NNR.0b013e318283f68e 23458910

